# ﻿Two novel freshwater hyphomycetes, in *Acrogenospora* (Minutisphaerales, Dothideomycetes) and *Conioscypha* (Conioscyphales, Sordariomycetes) from Southwestern China

**DOI:** 10.3897/mycokeys.101.115209

**Published:** 2024-01-31

**Authors:** Lu Li, Hong-Zhi Du, Vinodhini Thiyagaraja, Darbhe Jayarama Bhat, Rungtiwa Phookamsak, Ratchadawan Cheewangkoon

**Affiliations:** 1 Department of Entomology and Plant Pathology, Faculty of Agriculture, Chiang Mai University, Chiang Mai 50200, Thailand; 2 Innovative Agriculture Research Centre, Faculty of Agriculture, Chiang Mai University, Chiang Mai 50200, Thailand; 3 Key Laboratory for Plant Diversity and Biogeography of East Asia, Kunming Institute of Botany, Chinese Academy of Sciences, Kunming 650201, Yunnan Province, China; 4 School of Pharmacy, Guizhou University of Traditional Chinese Medicine, Guiyang 550025, Guizhou Province, China; 5 Department of Botany and Microbiology, College of Science, King Saud University, P.O. Box 2455, Riyadh 11451, Saudi Arabia; 6 Vishnugupta Vishwavidyapeetam, Ashoke, Gokarna 581326, India; 7 Honghe Center for Mountain Futures, Kunming Institute of Botany, Chinese Academy of Sciences, Honghe County 654400, Yunnan, China

**Keywords:** Acrogenosporaceae, Conioscyphaceae, freshwater fungi, new taxa, taxonomy

## Abstract

Freshwater fungi are highly diverse in China and frequently reported from submerged wood, freshwater insects, herbaceous substrates, sediments, leaves, foams, and living plants. In this study, we investigated two freshwater species that were collected from Yunnan and Guizhou provinces in China. Detailed morphological analysis complemented by multi-gene phylogenetic analyses based on LSU, SSU, ITS, *RPB2* and *TEF1-α* sequences data revealed them to be two new saprobic species, namely *Acrogenosporaalangii***sp. nov.** and *Conioscyphayunnanensis***sp. nov.** in their asexual morphs. Additionally, *Acrogenosporaalangii***sp. nov.** is reported for the first time as a freshwater ascomycete associated with the medicinal plant *Alangiumchinense* (Alangiaceae). Detailed morphological descriptions, illustrations and updated phylogenetic relationships of the new taxa are provided herein.

## ﻿Introduction

The freshwater fungi in China are taxonomically highly diverse which include members of Dothideomycetes, Eurotiomycetes, Laboulbeniomycetes, Leotiomycetes, Orbiliomycete, Pezizomycetes and Sordariomycetes (Hu et al. 2013; Calabon et al. 2022). The freshwater fungi are ecologically diverse, occurring on various substrates, including submerged wood, freshwater foams, herbaceous substrates, insects, leaves, sediments and other organic matter, and living plants (Hu et al. 2013; Shen et al. 2022; Calabon et al. 2023). Most species are well-known as saprobes and they play an important role in ecological functioning as decomposers but also can be pathogens as well as symbionts on humans and plants (Su et al. 2015; Su et al. 2016; Dong et al. 2020).

The order Minutisphaerales (Dothideomycetes) is known as the order for freshwater fungi and comprises two families, viz. Acrogenosporaceae and Minutisphaeraceae (Wijayawardene et al. 2022). Acrogenosporaceae was introduced by Jayasiri et al. (2018) to accommodate *Acrogenospora* based on morpho-molecular evidence. The genus *Acrogenospora* was introduced by Ellis (1971) for two species namely *A.sphaerocephala* (the type species), and *A.carmichaeliana* (as *Farlowiellacarmichaeliana*; asexual morph). A year later, Ellis (1972) added another new species, *A.setiformis*. While Goh et al. (1998) revised the genus and accepted eight species, Bao et al. (2020) re-investigated *Acrogenospora* and added seven new species that were reported from freshwater habitat. Subsequently, two new species *A.guizhouensis* and *A.stellata* were introduced in asexual and sexual states, respectively (Tan et al. 2022; Hyde et al. 2023). Presently, there are 23 epithets for *Acrogenospora* in Index Fungorum (http://indexfungorum.org/Names/Names.asp; accessed on 20 Nov. 2023).

*Acrogenospora* was considered as the asexual morph of *Farlowiella* which was further supported by the morpho-molecular analyses conducted by Jayasiri et al. (2018) and the pleomorphic status of these two genera was confirmed by Rossman et al. (2015) who recommended protecting the name *Acrogenospora* over *Farlowiella* based on the wider use and fewer name changes. The sexual morph of this genus is characterized by hysterothecial, thick-walled, apparently solitary to gregarious, but remaining erect and elevated and presenting an almost stipitate ascomata with a prominent sunken slit, 8-spored, cylindric-clavate, short pedicellate asci and 1–2-celled, hyaline or moderately pigmented ascospores (Sivanesan 1984; Boehm et al. 2009). The asexual morph is characterized by macronematous, mononematous, simple, brown, sometimes percurrently proliferating conidiophores; monoblastic, terminal or intercalary conidiogenous cells with globose, ellipsoid or obovoid, olivaceous to dark brown conidia (Hughes et al. 1978; Goh et al. 1998). The members of *Acrogenospora* mostly show similar morphology, but mainly distinguished by degree of pigmentation of the conidiophores, and conidial shape, size, color, guttules and basal cells (Hughes et al. 1978; Bao et al. 2020).

Conioscyphales (Sordariomycetes), a largely freshwater order, was introduced by Réblová et al. (2016) to accommodate a single family Conioscyphaceae and a genus *Conioscypha*. The order was placed within Hypocreomycetidae (Réblová et al. 2016). However, Conioscyphales clustered within the newly introduced subclass Savoryellomycetidae in the phylogenetic analyses conducted by Hongsanan et al. (2017). Höhnel (1904) had introduced *Conioscypha* with *C.lignicola* as the type species and the genus currently accommodates 18 species (Höhnel 1904; Matsushima 1975, 1993, 1996; Shearer 1973; Shearer and Motta 1973; Udagawa and Toyazaki 1983; Kirk 1984; Chen and Tzean 2000; Réblová and Seifert 2004; Crous et al. 2014, 2018; Zelski et al. 2015; Chuaseeharonnachai et al. 2017; Hernández et al. 2017; Feng and Yang 2018; Turland et al. 2018; Liu et al. 2019; Luo et al. 2019; Hyde et al. 2020; Jiang et al. 2022). Réblová and Seifert (2004) established *Conioscyphascus* based on *C.varius* which is the sexual morph of *Conioscyphavaria* and the sexual-asexual linkage was further confirmed by culture studies and molecular data (Réblová and Seifert 2004; Zelski et al. 2015). According to the nomenclatural priority, *Conioscyphascus* is synonymized under *Conioscypha* (Turland et al. 2018).

Species of *Conioscypha* are mostly reported from freshwater and terrestrial habitats and primarily recorded in their asexual morph. Only few species are reported in sexual morph (Shearer 1973; Shearer and Motta 1973; Kirk 1984; Zelski et al. 2015). The asexual morph is characterized by the enteroblastic percurrent conidiogenesis in distinct conidiogenous cells that retain successive wall layers at the same level as multi-collaretted as each conidium ruptures through the apex with dematiaceous aseptate conidia of various shapes (Shearer 1973; Shearer and Motta 1973; Kirk 1984; Zelski et al. 2015). The sexual morph is characterized by perithecial ascomata that are immersed to superficial, globose to subglobose, cylindrical-clavate asci with a pronounced non-amyloid apical annulus, transversely multi-septate and hyaline ascospores (Luo et al. 2019).

Guizhou and Yunnan provinces are mostly referred as part of the Southwestern China (Feng and Yang 2018; Jiang et al. 2022). This region is a center of biodiversity for freshwater fungi (Shen et al. 2022). Many new freshwater fungi have been reported in Yunnan and Guizhou provinces in recent years (Su et al. 2016; Wang et al. 2016; Li et al. 2017, 2020; Luo et al. 2018a, b, 2019; Zhao et al. 2018; Dong et al. 2020; Wan et al. 2021; Shen et al. 2022). In particular, Yunnan province stands out as a hotspot for freshwater fungal research (Luo et al. 2019; Dong et al. 2020; Shen et al. 2022). The diversity of freshwater fungi in streams and rivers in northwestern Yunnan has been intensely studied, resulting in the discovery of a large number of new species and new records in some highly diverse genera e.g. *Acrogenospora*, *Dictyosporium*, *Distoseptispora*, *Pleurotheciella*, *Sporidesmium* and *Sporoschisma* (Su et al. 2016; Wang et al. 2016; Li et al. 2017, 2020; Luo et al. 2018a, b, 2019; Zhao et al. 2018; Bao et al. 2020; Wan et al. 2021; Shen et al. 2022).

In this study, two collections were obtained from decaying submerged wood and dead branches of *Alangiumchinense* in freshwater habitat in Southwestern China. Multi-gene phylogenetic analyses based on Maximum likelihood (ML) and Bayesian analyses along with morphological characters support the establishment of the new species. We also provided a comparative synoptic table for *Conioscypha*. This study adds new data to our knowledge on fungal diversity of freshwater streams in Southwestern China.

## ﻿Materials and methods

### ﻿Sample collection, isolation and morphological studies

Submerged decaying wood and branches were collected from Guizhou and Yunnan provinces, China. Fresh specimens were studied following the methods described by Luo et al. (2018b). The samples were incubated in plastic boxes at room temperature for one week. Micromorphological characters were observed using a stereomicroscope (SteREO Discovery.V12, Carl Zeiss Microscopy GmBH, Germany) and photographed using a Nikon ECLIPSE 80i compound microscope fitted with a NikonDS-Ri2 digital camera. Microscopic structures were measured using Tarosoft (R) Image Frame Work program and the photomicrographs were processed using Adobe Photoshop CS6 version 10.0 software (Adobe Systems, USA).

Single spore isolation was performed following the method described by Luo et al. (2018b). The germinated conidia were transferred to fresh PDA plates and incubated at room temperature. The specimens were dried under natural light, wrapped in absorbent paper, and placed in a Ziplock bag with mothballs. Herbarium specimens were deposited in the Herbarium of Cryptogams, Kunming Institute of Botany Academia Sinica (**KUN-HKAS**), Kunming, China, and Herbarium, University of Electronic Science and Technology (**HUEST**), Chengdu, China. The cultures were deposited in Kunming Institute of Botany, Chinese Academy of Sciences (**KUNCC**), Kunming, Yunnan, China and the University of Electronic Science and Technology Culture Collection (**UESTCC**), Chengdu, China. The novel species were registered in Faceoffungi (Jayasiri et al. 2015) and MycoBank databases (https://www.mycobank.org/mycobank-deposit; accessed on 22 September 2023).

### ﻿DNA extraction, PCR amplification and sequencing

Fresh mycelia were scraped from colonies grown on potato dextrose agar (PDA) medium. DNA extraction was carried out using DNA extraction kit following the manufacturer’s instructions (TOLOBIO Plant Genomic DNA Extraction Kit, Tsingke Company, Beijing, P.R. China). PCR amplification was performed using primers pairs LR0R/LR5 (Vilgalys and Hester 1990) for the nuclear ribosomal large subunit 28S rDNA gene (LSU); NS1/NS4 (White et al. 1990) for the nuclear ribosomal small subunit 18S rDNA gene (SSU); ITS5/ITS4 (White et al. 1990) for the internal transcribed spacer rDNA region (ITS); fRPB2-5F/fRPB2-7cR (Liu et al. 1999) for the RNA polymerase second largest subunit (RPB2); and EF1-983F/EF1-2218R (Rehner and Buckley 2005) for the translation elongation factor 1-alpha (*TEF1-α*). The PCR amplification was carried out in a 25 μL reaction volume containing 12.5 μL of 2× Power Taq PCR Master Mix, 1 μL of each forward and reward primer (10 μM), 1 μL of genomic DNA template (30–50 ng/μL) and 9.5 μL of sterilized double-distilled water. Amplifications were carried out using the BioTeke GT9612 thermocycler (Tsingke Company, Beijing, P.R. China). The PCR amplification conditions for ITS, LSU, and SSU consisted of initial denaturation at 98 °C for 3 minutes, followed by 35 cycles of denaturation at 98 °C for 20 seconds, annealing at 53 °C for 10 seconds, an extension at 72 °C for 20 seconds, and a final extension at 72 °C for 5 minutes. The PCR amplification condition for *RPB2* consisted of initial denaturation at 95 °C for 5 minutes, followed by 40 cycles of denaturation at 95 °C for 1 minute, annealing at 52 °C for 2 minutes, an extension at 72 °C for 90 seconds, and a final extension at 72 °C for 10 minutes. The amplification condition for *TEF1-α* consisted of initial denaturation at 94 °C for 3 minutes, followed by 35 cycles of 45 seconds at 94 °C, 50 seconds at 55 °C and 1 minute at 72 °C, and a final extension period of 10 minutes at 72 °C. Quality of PCR products were checked using 1% agarose gel electrophoresis and distinct bands were visualized in gel documentation system (Compact Desktop UV Transilluminator analyzer GL-3120). The PCR products were purified and obtained Sanger sequences by Tsingke Company, Beijing, P.R. China.

### ﻿Sequence alignments and phylogenetic analyses

The newly generated sequences were subjected to the nucleotide BLAST search via NCBI (https://blast.ncbi.nlm.nih.gov/Blast.cgi; accessed on 1 September 2023) for searching the closely related taxa and confirming the correctness of the sequences. The closely related taxa of the novel species were retrieved from GenBank based on nucleotide BLAST (www.ncbi.nlm.nih.gov/blast/) searches and recent publications (Liu et al. 2019; Bao et al. 2020). Outgroups were selected based on recently published data (Liu et al. 2019; Bao et al. 2020) (Tables 1, 2). Multiple sequence alignments were aligned with MAFFT v.7 (http://mafft.cbrc.jp/alignment/server/index.html; accessed on 2 September 2023) and automatically trimmed using TrimAl (http://phylemon.bioinfo.cipf.es/utilities.html; accessed on 2 September 2023). A combined sequence dataset was performed with SquenceMatrix v.1.7.8 (Capella et al. 2009; Vaidya et al. 2011; Katoh and Standley 2013). Phylogenetic relationships of the new species were performed based on Maximum likelihood (ML) and Bayesian inference (BI) analyses.

**Table 1.** Taxon names, strain numbers and GenBank accession numbers of the ITS, LSU, SSU, *RPB2* and *TEF1-α* sequences used in the phylogenetic analyses. Newly generated sequences are highlighted in black bold font. The ex-type strains are indicated by superscript T. “–” stands for no sequence data in GenBank.

Taxon	Voucher/Culture	GenBank accession number				
		ITS	LSU	SSU	*RPB2*	*TEF1-α*
***Acrogenosporaalangii***	**KUNCC 23**–**14553**^T^	**OR557426**	**OR553807**	**OR553806**	**OR575924**	**OR575926**
	**UESTCC 23.0140**	**OR578817**	**OR574254**	**OR574239**	**OR575925**	**OR575927**
*Acrogenosporaaquatica*	MFLUCC 16–0949	–	MT340732	–	MT367160	MT367152
	MFLUCC 20–0097^T^	–	–	MT340743	MT367159	MT367151
*Acrogenosporabasalicellularispora*	MFLUCC 16–0992^T^	–	MT340729	–	–	–
*Acrogenosporacarmichaeliana*	CBS 206.36	–	MH867287	AY541482	–	–
	CBS 179.73	–	–	GU296148	–	–
	CBS 164.76	–	GU301791	GU296129	GU371748	GU349059
	FMR11021	HF677172	HF677191	–	–	–
*Acrogenosporaguttulatispora*	MFLUCC 17–1674^T^	–	MT340730	–	MT367157	–
*Acrogenosporaobovoidspora*	MFLUCC 18–1622^T^	–	MT340736	MT340747	MT367163	MT367155
*Acrogenosporaolivaceospora*	MFLUCC 20–0096^T^	–	MT340731	MT340742	MT367158	MT367150
*Acrogenosporasphaerocephala*	MFLUCC 16–0179	MH606233	MH606222	–	MH626448	–
*Acrogenosporasubmerse*	MFLUCC 18–1324^T^	–	MT340735	MT340746	MT367162	MT367154
*Acrogenosporasubprolata*	MFLUCC 18–1314	–	MT340739	MT340750	–	–
*Acrogenosporastellata*	AMI-SPL 1243	OP439740	OP439739	–	–	–
*Acrogenosporaterricola*	PS3565	ON176299	ON176305	ON176286	–	–
	PS3417	ON176288	–	–	–	–
	PS3610 ^T^	ON176304	ON176306	ON176287	–	–
*Acrogenosporathailandica*	MFLUCC 17–2396^T^	MH606234	MH606223	MH606221	MH626449	–
*Acrogenosporaverrucispora*	MFLUCC 20–0098	–	MT340737	MT340748	–	–
	MFLUCC 18–1617	–	MT340738	MT340749	MT367164	MT367156
*Acrogenosporayunnanensis*	MFLUCC 20–0099	–	MT340734	MT340745	MT367161	MT367153
	MFLUCC 18–1611^T^	–	MT340733	MT340744	–	–
*Minutisphaeraaspera*	DSM 29478^T^	NR_154621	NG_060319	NG_065059	–	–
*Minutisphaerajaponica*	HHUF30098^T^	NR_119419	NG_042338	NG_064840	–	–

**Table 2.** Taxon names, strain numbers and GenBank accession numbers of the LSU, ITS, SSU and *RPB2* sequences used in the phylogenetic analyses. The newly generated sequences are highlighted in black bold font. The ex-type strains are indicated by superscript T. “–” stands for no sequence data in GenBank.

Taxon	Voucher/Culture	Gene accession numbers			
		LSU	ITS	SSU	*RPB2*
*Conioscyphaaquatica*	MFLUCC 18–1333^T^	MK835857	MK878383	–	MN194030
*Conioscyphabambusicola*	JCM 7245^T^	NG059037	NR154660	–	–
*Conioscyphaboutwelliae*	CBS 144928^T^	LR025183	LR025182	–	–
*Conioscyphahoehnelii*	FMR 11592	KY853497	KY853437	HF937348	–
*Conioscyphajaponica*	CBS 387.84^T^	AY484514	–	JQ437438	JQ429259
*Conioscyphalignicola*	CBS 335.93	AY484513	–	JQ437439	JQ429260
*Conioscyphaminutispora*	FMR 11245^T^	KF924559	NR137847	HF937347	–
*Conioscyphanakagirii*	BCC77658^T^	KU509985	KY859266	KU509984	KU513952
	BCC77659	KU509987	KY859267	KU509986	KU513952
*Conioscyphaperuviana*	CBS 137657^T^	NG058867	–	–	–
*Conioscyphapleiomorpha*	FMR 13134^T^	KY853498	KY853438	–	–
*Conioscyphasubmerse*	MFLU 18–1639^T^	MK835856	MK878382	–	–
*Conioscyphatenebrosa*	MFLU 19–0688^T^	MK804508	MK804506	MK804510	MK828514
	MFLU 19–0687	MK804509	MK804507	MK804511	MK828515
*Conioscyphavaria*	CBS 602.70	MH871654	MH859868	–	–
	CBS 436.70	MH871548	MH859785	–	–
	CBS 604.70	MH871656	MH859869	–	–
	CBS 603.70	MH871655	–	–	–
*Conioscyphaverrucosa*	MFLUCC 18-0419^T^	MN061364	MN061350	MN061352	MN061668
***Conioscyphayunnanensis***	**KUNCC23**–**13319**^T^	**OR478379**	**OR234669**	**OR478381**	**OR487158**
	**KUNCC23**–**13172**	**OR478380**	**OR478183**	**OR478382**	**OR487157**
*Parafuscosporellagarethii*	BCC79986^T^	KX958430	OK135602	KX958428	–
*Parafuscosporellamoniliformis*	MFLUCC 15–0626^T^	KX550895	NR152557	NG063614	–

**Abbreviation**: AMI-SPL: Collection of A. Mateos & S. De la Peña, Azores, Terceira, Portugal; **BCC**: BIOTEC Culture Collection, Thailand; **CBS**: CBS-KNAW Fungal Biodiversity Centre, Utrecht, The Netherlands; **DSM**: Leibniz Institute DSMZ-German Collection of Microorganisms and Cell Cultures GmbH, Braunschweig, Science Campus Braunschweig-Süd, Germany; **FMR**: Facultat de Medicina i Ciencies de la Salut, Reus, Spain; **JCM**: Japan Collection of Microorganism, RIKEN BioResource Center, Japan; **HHUF**: Herbarium of Hirosaki University, Japan; **KUNCC**: Kunming Institute of Botany, Chinese Academy of Sciences Culture Collection, Kunming, Yunnan, China; **MFLU**: the herbarium of Mae Fah Luang University, Chiang Rai, Thailand; **MFLUCC**: Mae Fah Luang University Culture Collection, Chiang Rai, Thailand; **PS**: the R. L. Gilbertson Mycological Herbarium at the University of Arizona (MYCO-ARIZ); **UESTCC**: University of Electronic Science and Technology Culture Collection, Chengdu, China.

Maximum likelihood (ML) was performed by RAxML-HPC2 v.8.2.12 on the XSEDE (8.2.12) tool via the CIPRES Science Gateway (http://www.phylo.org/portal2; accessed on 4 September 2023) (Stamatakis 2006; Miller et al. 2015) following the default setting but adjusted by setting 1,000 bootstrap replications and GTRGAMMA model of nucleotide substitution.

The evolution model for the Bayesian inference (BI) analyses was performed using MrModeltest v2.3 (Ronquist et al. 2012). GTR+I+G was selected as the best-fit model for LSU, SSU, ITS, *RPB2* and *TEF1-α* dataset. Markov Chain Monte Carlo sampling (MCMC) was computed to estimate Bayesian posterior probabilities (BPP) using MrBayes v.3.2.7 (Ronquist et al. 2012). Six simultaneous Markov chains were run for random trees for 1,000,000 generations and trees were sampled every 200^th^ generation. The first 10% of the total trees were set as burn-in and were discarded. The remaining trees were used to calculate Bayesian posterior probabilities (BPP) in the majority rule consensus tree (when the final average standard deviation of split frequencies reached below 0.01). Phylograms were visualized using FigTree v1.4.0 (Rambaut 2006) and rearranged in Adobe Photoshop CS6 software (Adobe Systems, USA).

The newly generated sequences were deposited in GenBank (Tables 1, 2). The final alignment and phylogenetic tree was registered in TreeBASE (http://www.treebase.org/) under the submission ID: 30847 (*Acrogenospora*) and ID:30689 (*Conioscypha*).

## ﻿Results

### ﻿Phylogenetic analyses

**Figure 1.**
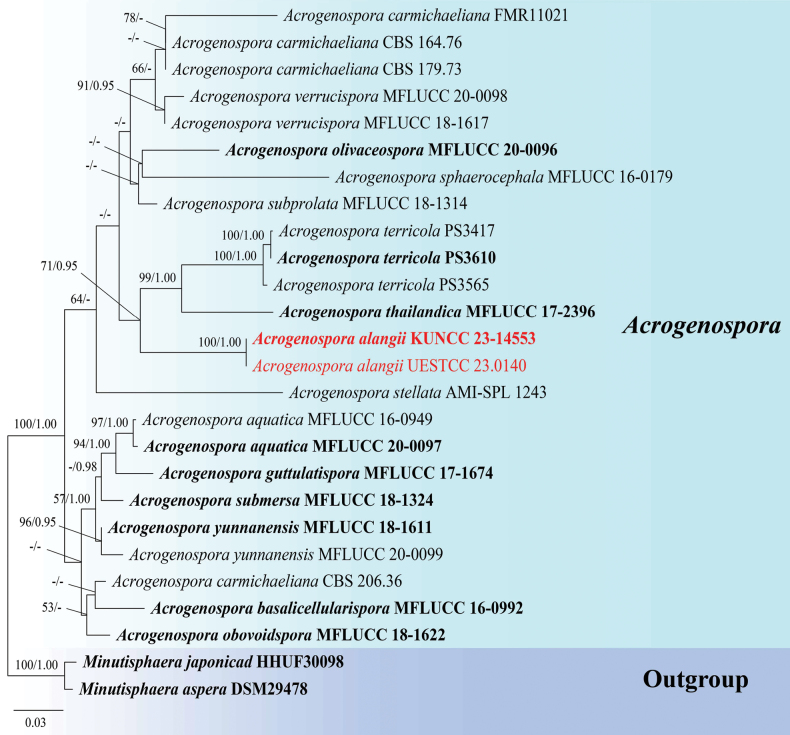
Phylogenetic tree constructed from RAxML analysis of LSU, SSU, ITS, *RPB2* and *TEF1-α* sequences data. Bootstrap support values for ML equal or greater than 50% and Bayesian posterior probabilities greater than 0.95 BPP are indicated at the nodes. The tree is rooted to *Minutisphaeraaspera* (DSM29478) and *Minutisphaerajaponica* (HHUF30098). The new isolates are in red bold.

Two phylogenetic analyses were conducted to resolve the phylogenetic affinities of the two new freshwater species, one each, within the genera *Acrogenospora* (Acrogenosporaceae/ Minutisphaerales/ Dothideomycetes; Analysis 1), and the other within *Conioscypha* (Conioscyphaceae/ Conioscyphales/ Sordariomycetes; Analysis 2), as follows:

**Analysis 1**: The phylogram generated from ML analysis based on combined LSU, SSU, ITS, *RPB2* and *TEF1-α* sequences data was selected to represent the relationship between the new species and other known species in *Acrogenospora*. Twenty-six strains were included in the combined dataset which comprised 4,527 characters (LSU: 987 bp, SSU: 1007 bp, ITS: 535 bp, *RRB2*: 1044 bp, *TEF1-α*: 954 bp) after alignment (including gaps). *Minutisphaeraaspera* (DSM29478) and *M.japonica* (HHUF30098) were selected as the outgroup taxa. The best RAxML tree with a final likelihood value of -15211.062629 is presented in Fig. 1. RAxML analysis yielded 1,028 distinct alignment patterns and 43.09% of undetermined characters or gaps. Estimated base frequencies were as follows: A = 0.260065, C = 0.232516, G = 0.268900, T = 0.238519, with substitution rates AC = 1.050467, AG = 3.191516, AT = 1.485302, CG = 1.086194, CT = 7.658416, GT = 1.000000; gamma distribution shape parameter alpha = 0.180026. The final average standard deviation of split frequencies at the end of total MCMC generations for BI analysis was 0.009674 (the critical value for the topological convergence diagnostic is below 0.01).

Phylogenetic analyses retrieved from ML and BI analyses were not significantly different and showed similar topologies. Phylogenetic analyses showed that our new collection (KUNCC23–14553 and UESTCC 23.0140) formed an independent subclade with strong statistical support (100% MLBS/ 1.00 BPP) and shared the same clade with *Acrogenospora.terricola* and *A.thailandica* with moderate statistical support (71% MLBS/ 0.95 BPP; Fig. 1).

**Figure 2.**
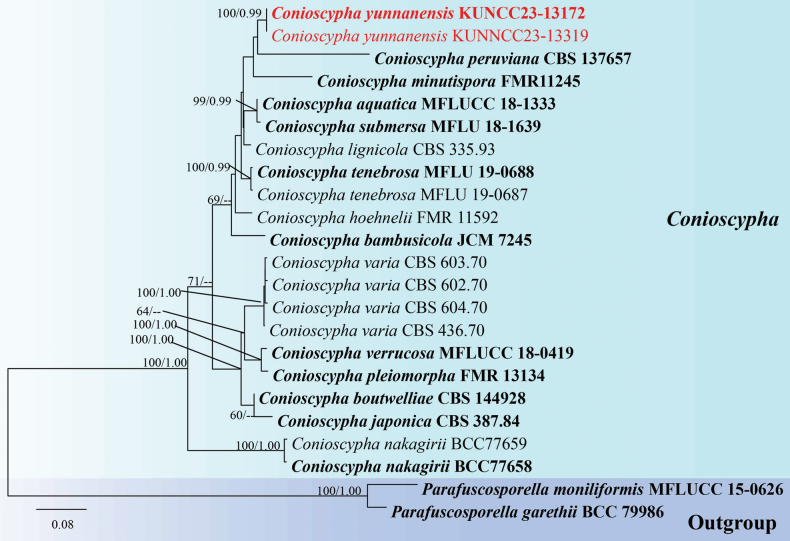
Phylogenetic tree constructed from RAxML analysis of LSU, ITS, SSU and *RPB2* sequences data. Bootstrap support values for ML equal or greater than 50% and Bayesian posterior probabilities greater than 0.95 BPP are indicated at the nodes. The tree is rooted to *Parafuscosporellamoniliformis* (MFLUCC 15–0626) and *P.garethii* (BCC79986). The new isolates are in red bold.

**Analysis 2**: The phylogram generated from ML analysis based on combined LSU, ITS, SSU and *RPB2* sequences data was selected to represent the relationship between the new species and other known species in *Conioscypha*. Twenty-three strains were included in the combined dataset which comprised 3,679 characters (LSU: 904 bp, ITS: 696 bp, SSU: 1026 bp, *RPB2*: 1053 bp) after alignment (including gaps). *Parafuscosporellagarethii* (BCC79986) and *P.moniliformis* (MFLUCC 15–0626) were selected as the outgroup taxa. The best RAxML tree with a final likelihood value of -14285.072957 is presented in Fig. 2. RAxML analysis yielded 1,112 distinct alignment patterns and 40.07% of undetermined characters or gaps. Estimated base frequencies were as follows: A = 0.236438, C = 0.267389, G = 0.295788, T = 0.200385, with substitution rates AC = 1.738303, AG = 2.933990, AT = 1.389088, CG = 1.593182, CT = 7.181256, GT = 1.000000; gamma distribution shape parameter alpha = 0.453781. The final average standard deviation of split frequencies at the end of total MCMC generations for BI analysis was 0.003901 (the critical value for the topological convergence diagnostic is below 0.01).

Phylogenetic analyses retrieved from ML and BI analyses were not significantly different and showed similar topologies. Phylogenetic analyses showed that our new collections (KUNCC 23–13319 and KUNCC 23–13172) formed an independent subclade with strong statistical support (100% MLBS/ 0.99 BPP) and clustered with *Conioscyphaperuviana* and *C.minutispora*. In this study, *C.aquatica* (MFLUCC 18–1333) shared the same branch length with *C.submersa* (MFLU 18-1636) with high statistic support (99% MLBS/ 0.99 BPP). Simultaneously, *C.pleiomorpha* (FMR 13134) shares the same branch length with *C.verrucosa* (MFLUCC 18–0419) with high support (100% MLBS/ 1.00 BPP). While *C.boutwelliae* (CBS 144928) also shares the same branch length with *C.japonica* (CBS 387.84), it exhibits low statistical support in both ML and BI analyses. Therefore, the conspecific status of these species is questionable.

### ﻿Taxonomy

#### ﻿ Acrogenospora alangii

Taxon classificationFungiMinutisphaeralesAcrogenosporaceae

H.Z. Du & Cheewangkoon sp. nov.

0F7A738A-820F-53F4-B43E-0F2BF63C1237

850015

Facesoffungi Number: FoF15041

Fig. 3

##### Etymology.

The epithet ‘*alangii*’ refers to the host genus *Alangium* on which the holotype was collected.

##### Holotype.

KUN-HKAS 130312.

##### Description.

***Saprobic*** on submerged decaying branches of *Alangiumchinense* (Alangiaceae). ***Asexual morph***: Hyphomycetous. ***Colonies*** on natural substrate, effuse, hairy, black, glistening. ***Mycelium*** partly semi-immersed, composed of septate, brown to dark brown, branched, smooth hyphae. ***Conidiophores*** 179–687 × 2.7–5.5 µm (*x*– = 485 × 4.2 µm, n = 20), mononematous, macronematous, solitary, erect, straight or slightly flexuous, cylindrical, unbranched, brown to dark brown, paler toward apex, septate, proliferating percurrently, smooth. ***Conidiogenous cells*** monoblastic, integrated, initially terminal, later becoming intercalary, cylindrical, smooth, pale brown. ***Conidia*** 15–22 × 15–23 µm (*x*– = 19.5 × 19 µm, n = 30) acrogenous, solitary, spherical or subspherical, truncate at base, aseptate, with apical appendages, hyaline and pale gray when young, pale to dark brown when mature, smooth. ***Sexual morph***: Undetermined.

##### Culture characteristics.

Conidia germinating on PDA within 24 h and germ tubes produced from the conidial base. Colonies reaching 16 mm diam at the room temperature in natural light for one month. Colonies on PDA medium dense, irregular in shape, slightly raised to umbonate or convex, surface rough, radially striated with lobate edge, fairy fluffy to floccose, white at the center, white-gray to gray sparse towards the margin; in reverse, white to white-gray at the center, with dark gray to brown-gray in the middle, white to pale yellowish at the edge, radiating outwards with irregular ring; no pigmentation on PDA.

**Figure 3.**
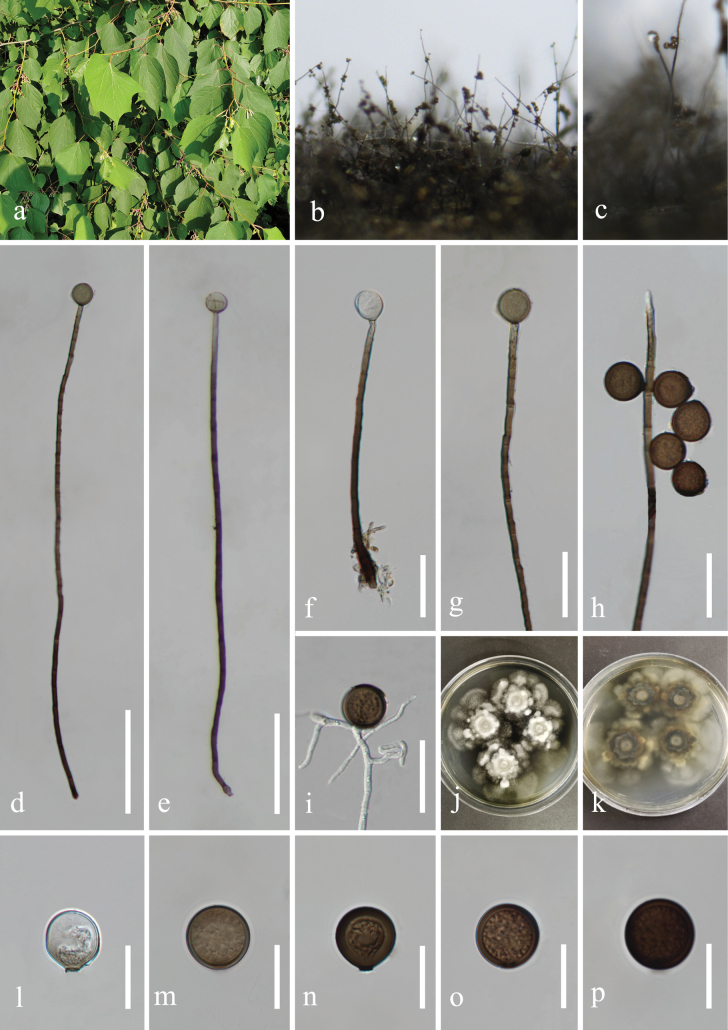
*Acrogenosporaalangii* (KUN-HKAS 130312, holotype) **a** hostplant growing near water body **b, c** colonies on host substrate **d–h** conidiophores, conidiogenous cells and conidia **i** germinating conidium **j, k** colony on PDA (up-front, down-reverse) **l, n** conidia with apical appendages **l–p** conidia. Scale bars: 100 µm (**d, e**), 40 µm (**f–i**), 20 µm (**l–p**).

##### Material examined.

China, Guizhou Province, Guiyang City, Wudang District, Xiangzhigou scenic spot, (26°46'7"N, 106°54′55"E), on dead branches of medicinal plant *Alangiumchinense* (Alangiaceae) from freshwater stream, 25 February 2022, H.Z. Du, S136 (KUN-HKAS 130312, ***holotype***), ex-holotype living culture = KUNCC 23–14553; ibid., S136A (HUEST 23.0140, isotype), ex-isotype living culture = UESTCC 23.0140.

##### Notes.

In the combined multi-locus phylogenetic analyses, *Acrogenosporaalangii* formed a distinct clade with *A.terricola* and *A.thailandica* with significant support (71% MLBS/ 0.95 BPP; Fig. 1). The nucleotide base pair comparison between *A.alangii* (KUNCC 23–14553) and *A.terricola* (PS 3610) revealed the differences as 25/829 bp (3.0%) of LSU and 5/1006 bp (0.50%) of SSU. While the differences between *A.alangii* (KUNCC 23–14553) and *A.thailandica* (MFLUCC 17–2396) showed 30/834 bp (3.6%) of LSU and 2/1029 bp (0.2%) of SSU and 131/1045 bp (12.5%) of *RPB2*. *Acrogenosporaalangii* can be distinguished from *A.terricola* in having conidia that are hyaline to pale gray when young, becoming pale brown to dark brown when mature, while *A.terricola* has olive green to dark brown conidia. Additionally, *A.thailandica* differs from *A.alangii* in having deep brown to black conidia (Hyde et al. 2019; Harrington et al. 2022). Furthermore, *A.alangii* also differs from the type species *A.sphaerocephala* in conidial color which is dark reddish brown, or pale to mid brown in *A.sphaerocephala* (Hughes 1978). Both *A.alangii* and *A.guizhouensis* were collected from Guizhou Province. However, morphological comparison of *A.alangii* with *A.guizhouensis* shows their differences in conidial color (hyaline, to pale gray, becoming pale brown to dark brown vs. brown) and position of conidial development (acropleurogenous vs. acrogenous) (Hyde et al. 2023).

#### ﻿ Conioscypha yunnanensis

Taxon classificationFungiConioscyphalesConioscyphaceae

L. Li, Bhat & Phookamsak sp. nov.

CD29EA67-3927-5CA7-979D-F2FD35881344

849830

Facesoffungi Number: FoF14746

Fig. 4

##### Etymology.

The specific epithet “*yunnanensis*” refers to the name of the region, Yunnan Province (China), from where the holotype was collected.

##### Holotype.

KUN-HKAS 129616.

##### Description.

***Saprobic*** on submerged wood and unidentified twigs from freshwater habitat. ***Asexual morph***: Hyphomycetous. ***Colonies*** on natural substrates effuse, black, glistening. ***Conidiophores*** reduced to conidiogenous cells. ***Conidiogenous cells*** phialidic, integrated, terminal, globose to subglobose, cup-shaped, percurrently proliferating in the same level, becoming multi-layered, multi-collaretted with outwardly curved edge, hyaline, smooth-walled. ***Conidia*** 18–26 × 17–22 µm (*x*– = 22 × 20 µm, n = 20), acrogenous, brown to dark brown, globose to subglobose, smooth-walled, aseptate, rounded at apex, subtruncate at base. ***Sexual morph***: Undetermined.

##### Culture characteristics.

Conidia germinating on PDA within 48 h and germ tubes produced from the conidial base. Colonies reaching 4.3 mm diam at room temperature in natural light for three months. Colonies on PDA medium dense to dense, circular, white and gray in the center, with packed mycelium, becoming black mycelial patch in the middle, white to cream at the margin, slightly radiating with irregular edge, radially furrowed aspect; in reverse, dark brown to black at the center, radiated with pale yellowish and dark greenish furrowed ring, white to cream at the margin with furrows aspects; no pigmentation on PDA.

**Figure 4.**
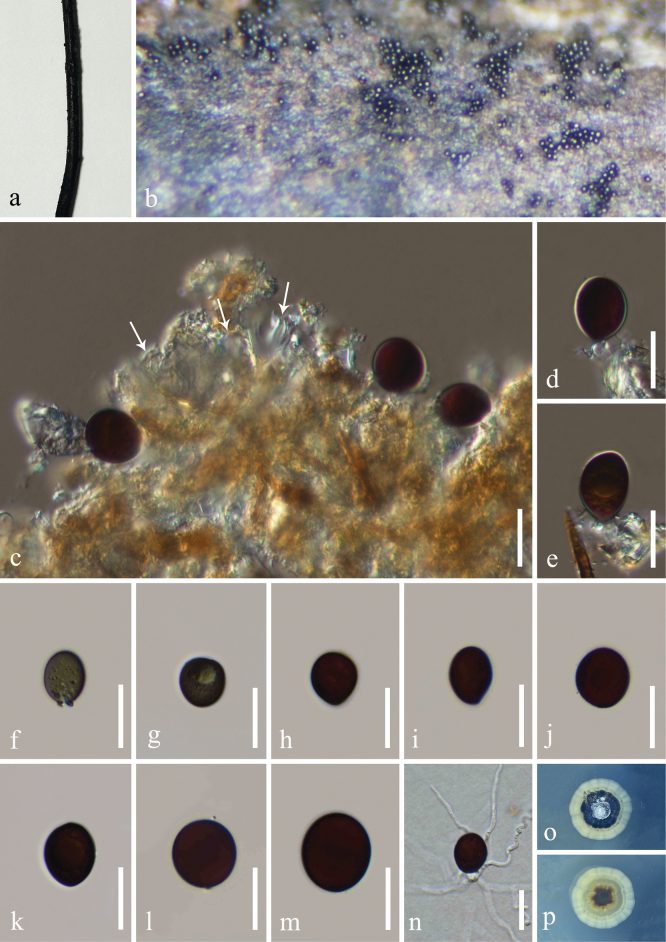
*Conioscyphayunnanensis* (KUN-HKAS 129616, holotype) **a** host specimen **b** colonies on submerged wood **c** conidiogenous cells bearing conidia (note: arrow points = cupulate conidionenous cells) **d, e** conidiogenous cell attached with conidia **f–m** conidia **n** germinated conidium **o, p** colony on PDA (**o** = up-front, **p** = down-reverse). Scale bars: 20 μm (**c–n**).

##### Material examined.

China, Yunnan Province, Xishuangbanna (21°10'–22°40'N, 99°55'–101°50'E), on decaying submerged wood in a freshwater stream, 9 September 2022, L. Li, LILU-117-1 (KUN-HKAS 129616, ***holotype***), ex-type living culture = KUNCC 23–13319; Dujuanhu Lake (22°29'–25°30'N, 100°16'–103°16'E), on unidentified twigs, 26 August 2022, LILU-109-1 (KUN-HKAS 129617, paratype), living culture KUNCC = 23–13172.

##### Notes.

*Conioscyphayunnanensis* has close phylogenetic relationships with *C.peruviana* and *C.minutispora*. The nucleotide base pair comparison between *C.yunnanensis* (KUNCC 23–13319) and *C.peruviana* (CBS 137657) revealed 95/828 bp (11.2%) of LSU differences. The nucleotide base pair comparison between *C.yunnanensis* (KUNCC 23–13319) and *C.minutispora* (FMR 11245) revealed 57/623 bp (9.2%) of LSU, 118/554 bp (22%) of ITS and 10/937 (1.1%) of SSU differences. The new taxon shares similar morphology with *C.peruviana* in having cup-like phialidic conidiogenous cells, and brown conidia but differing by varied shapes (globose to subglobose vs. ellipsoidal to allantoid or fabiform), the size (18–26 × 17–22 µm vs. 13.5–18 × 5–8.5 µm) and absence of lipid droplets (Zelski et al. 2015). *Conioscyphayunnanensis* also resembles *C.minutispora* in having subglobose conidia but differs in the size measurement (18–26 × 17–22 µm vs. 6–9 × 5–6 µm) (Crous et al. 2014). Furthermore, *C.yunnanensis* shares similar morphology to the type species *C.lignicola* in having micronematous conidiophores and globose to subglobose conidia that are brown. However, *C.yunnanensis* differs by the absence of dark brown ring surrounded in the conidia and presence of guttules periphery of conidia (Shearer 1973). The morphological comparison with other *Conioscypha* species is also provided in Table 3.

**Table 3.** Synopsis and distribution of *Conioscypha* species. The new species is indicated by black bold.

Species	Conidiophores	Conidiogenous cells	Conidia	Hosts	References
				Habitats	
				Distribution	
*Conioscyphaaquatica*	–	–	Globose to subglobose, dark brown to black, 19–23 × 17–21 μm	Submerged wood	Luo et al. 2019
				Freshwater	
				China	
*C.bambusicola*	Semi-macronematous to micronematous, mononematous	Percurrent, cuneiform, 1.6–8.0 × 2.3–4.8 μm	Ovoid or broadly obclavate, base truncate, apex apiculate, dark brown, 11–16 × 6–10 µm	Bamboo	Matsushima 1975
				Terrestrial	
				Japan	
*C.boutwelliae*	Reduced to conidiogenous cells	Monoblastic, endogenous, 11.5–20.5 × 8–15 µm	Ellipsoidal, obovoid or subglobose, base truncate with a central pore of 1–1.5 μm diam, brown, pitted-wall, 10.5–21 × 8–13.5 µm	Soil	Crous et al. 2018
				Terrestrial	
				Netherlands	
*C.dimorpha*	–	–	Macroconidia: oblong to cylindrical, apex round, base truncate, olivaceous to brown, 8–18 × 4–6.5 µm	Decayed leaves	Matsushima 1996
			Microconidia: subglobose to oblong, apex round, base truncate, pale brown, 2.0–3.0 × 2.0–2.5 µm	Terrestrial	
				South Africa	
*C.fabiformis*	–	–	Oblong or round, slightly curved, olivaceous, black in mass, 10–16 × 4.5–6.6 µm	Decayed leaves	Matsushima 1993
				Terrestrial	
				Peru	
*C.gracilis*	–	–	Ellipsoidal to flammiform, base truncate, slightly tapering towards apex, reddish brown, 8.5–9.5 × 5.5–7 µm, L/W 1.6:1	Decayed wood	Zelski et al. 2015
				Terrestrial and Freshwater	
				Denmark and Japan	
*C.hoehnelii*	Semi-macronematous to micronematous, mononematous	Cuneiform, cylindrical, often with a conspicuous cup-shaped, multi-collarette at the apex	Globose to subglobose or sometimes irregular, with a central pore in the inconspicuous scar at the base, brown to dark brown, 12–17 × 11–15 µm	Bark of *Eucalyptus* sp., leaf of *Phormiumtenax* and unidentified wood	Kirk 1984; Chen and Tzean 2000
				Terrestrial	
				UK and China	
*C.japonica*	Micronematous to semi-macronematous, mononematous	Percurrent, with a multi-layered, cup-like, collarette at the apex, 4.0–17.6 × 3.2–3.8 µm	Obpyriform or subglobose, sometimes elongate, base truncate, broadly rounded at apex, smooth but with irregular pigments deposited at the periphery of the wall to give the appearance of roughness, with a pore at the point of attachment to the conidiogenous cell, entirely covered by a thin gelatinous sheath, dark brown, 9–14 × 4.5–10 µm	Scraping and hair of male dog and rotten herbaceous stem	Udagawa and Toyazaki 1983; Chen and Tzean 2000
				Terrestrial	
				Japan and China	
*C.lignicola*	Micronematous to semi-macronematous, mononematous	Mostly cuneiform, doliiform, percurrent, often with a cup-shaped multi-collarette, up to 16.0 µm wide at the apex, 1.6–4.8 × 2.8–6.8 µm	Obovate or sometimes subglobose, truncate at the base, with reduced lumina, smooth but dark dots deposited at the periphery, at the base with a central pore, surrounded by a dark brown ring, 11–21.6 × 10.6–16.8 µm	Balsa wood and rotten leaf of *Phyllostachyspubescens*	Shearer and Motta 1973; Shearer 1973; Chen and Tzean 2000
				Freshwater and terrestrial	
				USA and China	
*C.minutispora*	Reduced to conidiogenous cells	Cuneiform, percurrent, with a cup-like collarette, up to 4.0 µm wide at the apex, 7–10 × 4–5 µm	Ellipsoidal, obovoid or subglobose, apex rounded, base truncate with a central pore, dark brown, 6–9 × 5–6 µm	Submerged wood	Crous et al. 2014
				Freshwater	
				Spain	
*C.nakagirii*	Micronematous to semi-macronematous, mononematous	Cuneiform, cylindrical, percurrent, with a cup-shaped multi-collarette, up to 50 µm wide at the apex, 7.5–15 × 5–7.5 µm	Turbinate to pyriform, rounded at apex, truncate with a basal pore	Submerged wood	Chuaseeharonnachai et al. 2017
				Freshwater	
				Thailand	
*C.peruviana*	–	–	Ellipsoidal to allantoid or fabiform, containing lipid droplets, brown, 13.5–18 × 5–8.5 μm	Submerged wood	Zelski et al. 2015
				Freshwater	
				Peru	
*C.pleiomorpha*	Micronematous, reduced to conidiogenous cells	Monoblastic, cupulate, endogenous, multilayer-cupulate collarette after several percurrent, enteroblastic, tiny elongations, 9–12 × 13–16 μm, up to 14.0 μm	Ellipsoidal, obovoid or subglobose, base truncate with a central pore, brown, 13–18 × 12–14 μm	Dead wood	Hernández-Restrepo et al. 2017
				Unknown habitat	
				Spain	
*C.submersa*	Reduced to conidiogenous cells	–	Globose to subglobose or ovoid, pale brown, guttulate, when young, dark brown to black when mature, 17–19 × 15–17 μm	Submerged wood	Luo et al. 2019
				Freshwater	
				China	
*C.tenebrosa*	Micronematous, mononematous, often reduced to conidiogenous cells	Phialidic, integrated, sessile or on short conidiophores, subcylindrical, percurrently proliferating, with cup-shaped multi-collarette	Globose to subglobose, obovoid, olivaceous, aseptate, broadly rounded at apex, base subtruncate, dark brown to black when mature, 18–25 × 14–20 μm	Submerged wood	Liu et al. 2019
				Freshwater	
				China	
*C.taiwaniana*	Micronematous to semi-macronematous, mononematous	Cuneiform, percurrent, smooth, hyaline, with a multilayered cup-like collarette, up to 25.0 µm wide at the apex, 2.8–6.4 × 4.0–7.2 µm	Ovoid or broadly obclavate, truncate at the base, often tapering towards a point at the apex, olive brown to yellowish brown or dark brown, 14.1–20.0 × 6.4–8.0 µm	Decaying stem	Hyde et al. 2020
				Terrestrial	
				China	
*C.varia*	–	–	Ovoid, flammiform, naviculiform, or subellipsoid, dark brown, 8.4–15 × 5.6–8.5 µm	Balsa wood	Shearer and Motta 1973; Shearer 1973
				Freshwater	
				USA	
*C.verrucosa*	Macronematous, mononematous, sometimes reduced to conidiogenous cells	Monoblastic, integrated, terminal, globose to ellipsoidal, 5.5–13 × 5–11.5 μm	Globose, subglobose, ellipsoidal or obovoid, aseptate, verrucose, guttulate, dark olivaceous to with a central basal pore, dark olivaceous to dark brown, 12.5–23 × 10.5–20 μm	Submerged wood	Hyde et al. 2020
				Freshwater	
				China	
***C.yunnanensis***	**Reduced to conidiogenous cells**	**Monoblastic, phialidic, integrated, terminal, globose to subglobose, cup-shaped, percurrently proliferating to the same level, multi-collarette with outwardly curved edge, hyaline, smooth-walled**	**Globose, subglobose, smooth-walled, aseptate, rounded at apex, subtruncate at base, brown to dark brown, 18–26 × 17–22 µm**	**Submerged wood**	**This study**
				**Freshwater**	
				**China**	

## ﻿Discussion

Dothideomycetes and Sordariomycetes are the two largest classes of lignicolous freshwater fungi (Luo et al. 2019; Calabon et al. 2022; Shen et al. 2022). In this study, two new freshwater species belonging to Dothideomycetes and Sordariomycetes were introduced which add to the fundamental knowledge on the diversity of freshwater fungi in Southwestern China. Furthermore, an updated phylogenetic information was provided and thus we attempted to resolve the taxonomic ambiguities of the genus *Acrogenospora* (Acrogenosporaceae, Minutisphaerales, Dothideomycetes) and *Conioscypha* (Conioscyphaceae, Conioscyphales, Sordariomycetes). The study will provide a better understanding of the taxonomic boundaries of these two genera with the illustration of two new species.

*Acrogenospora* species are mostly reported from freshwater habitats (Bao et al. 2020; Hyde et al. 2023). Recent studies have revealed that more than half of the new and interesting *Acrogenospora* species were observed from freshwater habitats in China, including *A.alangii* (in this study), *A.aquatica*, *A.basalicellularispora*, *A.ellipsoidea*, *A.guizhouensis*, *A.guttulatispora*, *A.hainanensis*, *A.obovoidspora*, *A.olivaceospora*, *A.ovalia*, *A.sphaerocephala*, *A.submersa*, *A.subprolata*, *A.verrucispora* and *A.yunnanensis* (Goh et al. 1998; Ho et al. 2001; Zhu et al. 2005; Hu et al. 2010; Bao et al. 2020; Hyde et al. 2023). Previous studies revealed that the highest number of *Acrogenospora* species were reported from Yunnan Province whereas a few species have been reported from Guizhou, Hainan, Hongkong, and Xizang. This suggests a high diversity of freshwater fungi in Yunnan Province, especially of the genus *Acrogenospora*. Simultaneously, Guizhou Province is located in southwestern China that shares similar biogeographical environments with Yunnan Province and therefore, the province may also offer a potential diversity of *Acrogenospora*.

Morphologically, species of *Acrogenospora* are distinguished from each other with difficulty and previous studies made efforts to segregate them based on shape, size, and color of the conidia and the degree of pigmentation of the conidiophores (Hughes 1978; Bao et al. 2020). A comprehensive study of *Acrogenospora* was carried out by Bao et al. (2020) who provided an updated taxonomic treatment of *Acrogenospora* and introduced seven new *Acrogenospora* species from Yunnan, China. Bao et al. (2020) and Hughes (1978) also provided a synoptic table of morphological comparison for all known *Acrogenospora* species. No significant morphological differences have been observed among known *Acrogenospora* species according to the species delineation provided by Bao et al. (2020). However, phylogenetic evidence and nucleotide pairwise comparison provide adequate justification for our species novelty following the recommendation of Jeewon and Hyde (2016).

The host association of freshwater fungi is difficult to identify. Besides, *Acrogenospora* species were mostly reported on submerged wood. Interestingly, the host associations of some *Acrogenospora* species (e.g., *A.altissima*, *A.gigantospora*, *A.sphaerocephala*, and *A.verrucispora*) have been identified. In this study, we reported *A.alangii* from freshwater habitat and associated with the medicinal plant *Alangiumchinense* for the first time.

Preliminary phylogenetic analyses of a combined LSU, SSU, ITS, *RPB2* and *TEF1-α* sequence dataset based on Maximum likelihood (ML) (Suppl. material 1: fig. S1) showed that *Acrogenospora* sp. (JX 43) [as *Farlowiellacarmichaeliana*] is sister to *A.submersa* (MFLUCC 18–1324) with low support in this study. Hyde et al. (2019) identified *Acrogenospora* sp. (JX 43) as *A.thalandica* based on phylogenetic evidence of a combined LSU, SSU and ITS sequence dataset. However, we have rechecked the sequences of *Acrogenospora* sp. (JX 43) via NCBI nucleotide BLAST search. The nucleotide BLAST search of LSU (KF836062) showed the similarity of this strain to *Chaetomiumglobosum* (CBS 828.73) with 100% similarity (Identities: 894/894 bp with no gap), of SSU (KF836061) showed 100% similarity to *A.thailandica* (MFLUCC 17–2396) (Identities: 1025/1025 bp with no gap), and of ITS (KF836060) showed 96.31% similarity to *Camposporiumcambrense* (CBS 132486) (Identities: 496/515 bp with 5 gaps). As the nucleotide BLAST results showed three different genes of *Acrogenospora* sp. (JX 43) aligning in different genera, we excluded this strain from our analysis to avoid misidentification.

Present phylogenetic analyses also showed that *Acrogenosporacarmichaeliana* (CBS 206.36) formed a separated clade with other strains of *A.carmichaeliana* (CBS 164.76, CBS 179.73, FMR 11021). *Acrogenosporacarmichaeliana* (CBS 206.36) was identified as *Farlowiellacarmichaeliana* (sexual morph) by E.W. Mason (https://wi.knaw.nl/fungal_table; accessed on 17 October 2023). While strain CBS 164.76 was priorly identified as *Acrogenosporasphaerocephala* (on decaying wood in Belgium), strain CBS 179.73 was identified as *Farlowiellacarmichaeliana* (on decaying wood in Germany) and FMR 11021 was identified as *Farlowiellacarmichaeliana* (unknown source). Hyde et al. (2019) introduced a new species *A.thailandica* and designated the reference specimen for the type species of *Acrogenospora*, *A.sphaerocephala*. Based on their phylogenetic analyses, these four unpublished strains were identified as *A.carmichaeliana*. However, the molecular data from the ex-type strain of *A.carmichaeliana* is unavailable. Therefore, the phylogenetic affinity of *A.carmichaeliana* remains uncertain, pending further study.

According to current reports, the species of *Conioscypha* are distributed worldwide, including Africa (*C.dimorpha*) (Matsushima 1996), United States of America (*C.lignicola*, *C.fabiformis*, *C.peruviana* and *C.varia*) (Matsushima 1993; Shearer 1973; Shearer and Motta 1973; Chen and Tzean 2000; Zelski et al. 2015), Asia (*C.aquatica*, *C.bambusicola*, *C.gracilis*, *C.hoehnelii*, *C.japonica*, *C.lignicola*, *C.nakagirii*, *C.submersa*, *C.tenebrosa*, *C.taiwaniana*, *C.verrucosa* and *C.yunnanensis*) (Shearer 1973; Shearer and Motta 1973; Matsushima 1975; Udagawa and Toyazaki 1983; Kirk 1984; Chen and Tzean 2000; Zelski et al. 2015; Chuaseeharonnachai et al. 2017; Liu et al. 2019; Luo et al. 2019; Hyde et al. 2020) and Europe (*C.gracilis*, *C.boutwelliae*, *C.hoehnelii*, *C.minutispora* and *C.pleiomorpha*)(Kirk 1984; Chen and Tzean 2000; Crous et al. 2014, 2018; Zelski et al. 2015; Hernández et al. 2017). In China, so far nine species have been reported including *C.aquatica*, *C.hoehnelii*, *C.japonica*, *C.lignicola*, *C.submersa*, *C.taiwaniana*, *C.tenebrosa* and *C.verrucosa* (Shearer 1973; Shearer and Motta 1973; Udagawa and Toyazaki 1983; Kirk 1984; Chen and Tzean 2000; Liu et al. 2019; Luo et al. 2019; Hyde et al. 2020).

Through our research on *Conioscyphayunnanensis*, it has been observed that the species of *Conioscypha* are largely indistinguishable in morphology. Hence, it has become necessary to use the potential of phylogenetic markers for clarifying their phylogenetic relationships. In this study, the single gene trees of *Conioscypha* (ITS, LSU, SSU, *RPB2*) and combined sequence datasets (LSU-ITS, LSU-ITS-SSU, and LSU-ITS-*RPB2*) were priorly conducted for comparing the reliable phylogenetic markers (Suppl. material 1: figs S2–S8). The results of these prior analyses demonstrated that the addition of *RPB2* gene could provide a better phylogenetic resolution of *Conioscypha*. Therefore, *RPB2* gene is recommended as a genetic marker for resolving phylogenetic relationships among species in *Conioscypha*.

Present phylogenetic analyses indicated that our new species formed a stable subclade independently and clustered with *Conioscyphaperuviana* (CBS 137657, ex-type strain) and *C.minutispora* (FMR 11245, ex-type strain). However, the phylogenetic relationships of these three species are not well-resolved in the present study. This may be due to the available sequence data wherein only LSU gene is available for *C.peruviana* while ITS, LSU, and SSU sequences are available for *C.minutispora*. Due to the recommendation of using *RPB2* gene for delineating species of *Conioscypha*, more sequence data of *C.peruviana* and *C.minutispora* are required for providing a better phylogenetic resolution on *Conioscypha*.

Meanwhile, *Conioscyphaaquatica* and *C.submersa*, introduced by Luo et al. (2019), were shown to be conspecific in the present phylogenetic analyses. Comparison of nucleotide pairwise of ITS and *TEF1-α* also demonstrated that these two species were not significantly different (7/530 bp (1.32%) of ITS and 10/801 bp (1.24%) of *TEF1-α*). However, *C.submersa* lacks *RPB2* gene that could separate these two species. Therefore, we tentatively instate these two species as a distinct species until the reliable gene (*RPB2*) is analyzed for resolving their conspecific status. Simultaneously, *C.pleiomorpha* and *C.verrucosa* have also been shown to be conspecific in the present phylogenetic analyses. However, a comparison of nucleotide pairwise of ITS and LSU demonstrated that these two species are different in 24/515 bp (4.66%) of ITS and 7/852 bp (0.82%) of LSU which is adequate to justify the species’ novelty. Similarly, there are only ITS and LSU sequence data of *C.pleiomorpha* currently available. These two genes may be inadequate to resolve the phylogenetic relationship of *C.pleiomorpha* and *C.verrucosa*.

The phylogenetic relationship of *Conioscyphaboutwelliae* and *C.japonica* is not well-resolved in the present study. This also may be affected by the available genes used in the analyses. There are only ITS and LSU sequences available for *C.boutwelliae* whereas LSU, SSU, and *RPB2* are available for *C.japonica*. Unfortunately, the nucleotide pairwise comparison between *C.boutwelliae* and *C.japonica* could not be done due to the LSU sequence of *C.japonica* is too short (531 bp) and lacking needful genetic information compared with *C.boutwelliae* (1,053 bp). Notably, *C.boutwelliae* was introduced by Crous et al. (2018). The species was isolated from soil in the Netherlands (holotype CBS H-23743, cultures ex-type CBS 144928 = JW203008, GenBank no. LR025182 (ITS) and LR025183 (LSU), MycoBank: MB828023). The search results of LR025182 (ITS) and LR025183 (LSU) via NCBI nucleotide search brought us to the species *C.pleiomorpha*. We have rechecked the detailed source information of LR025182 (ITS) and LR025183 (LSU) and resolved that the source information belongs to *C.boutwelliae*, resulting that the sequence name of *C.boutwelliae* is incorrect in NCBI database and the name “*C.boutwelliae*” should instead be referred to as “*C.pleiomorpha*” for the GenBank no. LR025182 (ITS) and LR025183 (LSU).

## Supplementary Material

XML Treatment for Acrogenospora alangii

XML Treatment for Conioscypha yunnanensis

## ﻿Additional information

### Author ORCIDs

Lu Li https://orcid.org/0000-0003-4695-2528

Hong-Zhi Du https://orcid.org/0000-0003-0350-4530

Vinodhini Thiyagaraja https://orcid.org/0000-0002-8091-4579

Darbhe Jayarama Bhat https://orcid.org/0000-0002-3800-5910

Rungtiwa Phookamsak https://orcid.org/0000-0002-6321-8416

Ratchadawan Cheewangkoon https://orcid.org/0000-0001-8576-3696

### Data availability

All of the data that support the findings of this study are available in the main text or Supplementary Information.

## Supplementary materials

Supplementary material 1 Supplementary document Data type docx https://binary.pensoft.net/file/976820This dataset is made available under the Open Database License (http://opendatacommons.org/licenses/odbl/1.0/). The Open Database License (ODbL) is a license agreement intended to allow users to freely share, modify, and use this Dataset while maintaining this same freedom for others, provided that the original source and author(s) are credited.Lu Li, Hong-Zhi Du, Vinodhini Thiyagaraja, Darbhe Jayarama Bhat, Rungtiwa Phookamsak, Ratchadawan Cheewangkoon
