# Supplementary material for: ﻿Two novel freshwater hyphomycetes, in Acrogenospora (Minutisphaerales, Dothideomycetes) and Conioscypha (Conioscyphales, Sordariomycetes) from Southwestern China
Source: MycoKeys. 2024 Jan 31;101:249–73. doi: 10.3897/mycokeys.101.115209 (PMC10851161; doi:10.3897/mycokeys.101.115209)
Supplement: Supplementary material 1 — Supplementary document [file mycokeys-101-249-s001.docx]

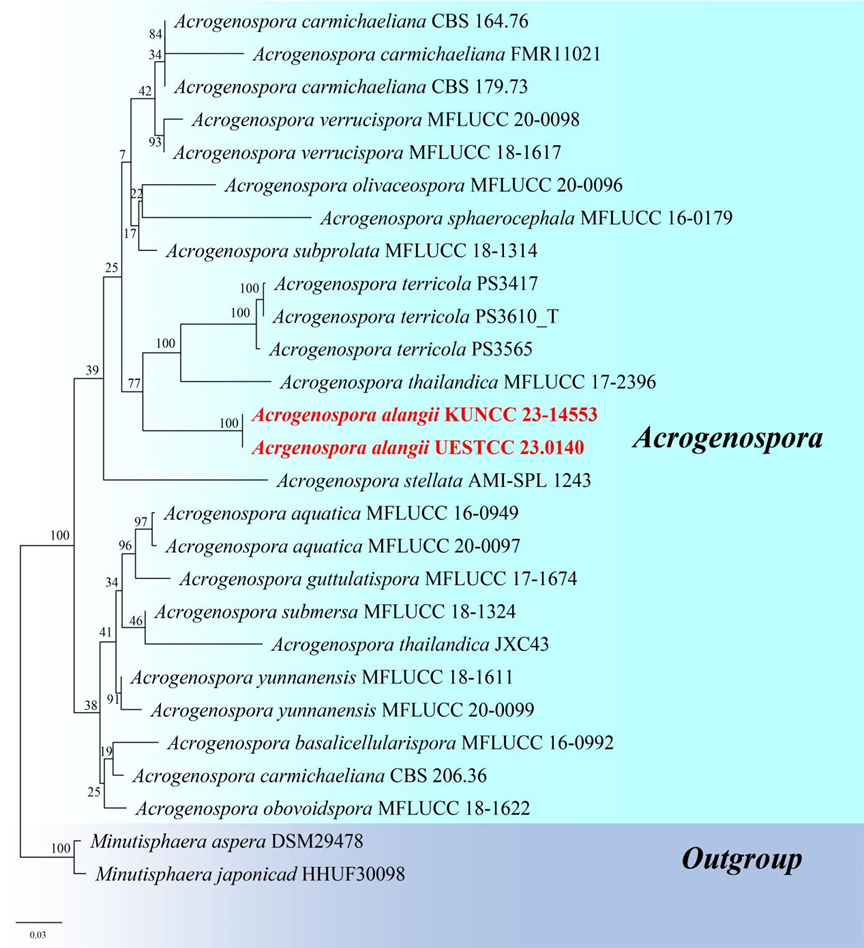


Figure 1. shows the phylogram generated from ML analysis based on combined LSU, SSU, ITS, *RPB2* and *TEF1-α* sequences data was selected to represent the relationship between the new species and other known species in *Acrogenospora*. Twenty seven strains are included in the combined dataset which comprised 4531 characters (LSU: 987 bp, SSU: 1009 bp, ITS: 537 bp, *PRB2*: 1044 bp, *TEF1-α*: 954 bp) after alignment. The best RAxML tree with a final likelihood value of -15681.568690 is presented. RAxML analysis yielded 1062 distinct alignment patterns and 44.01% of undetermined characters or gaps. Estimated base frequencies were as follows: A = 0.260420, C = 0.231911, G = 0.268330, T = 0.239340, with substitution rates AC = 1.000353, AG = 2.888284, AT = 1.379835, CG = 1.030636, CT = 6.990481, GT = 1.000000; gamma distribution shape parameter alpha = 0.179865. Phylogenetic analyses also showed that the new taxon was formed an independent subclade with strong statistical support (100%MLBDS /1.00 PP) and clustered with A. terricola and A. thailandica. Selecte Minutisphaera aspera (DSM29478) and M. japonicad (HHUF30098) as outgroup.


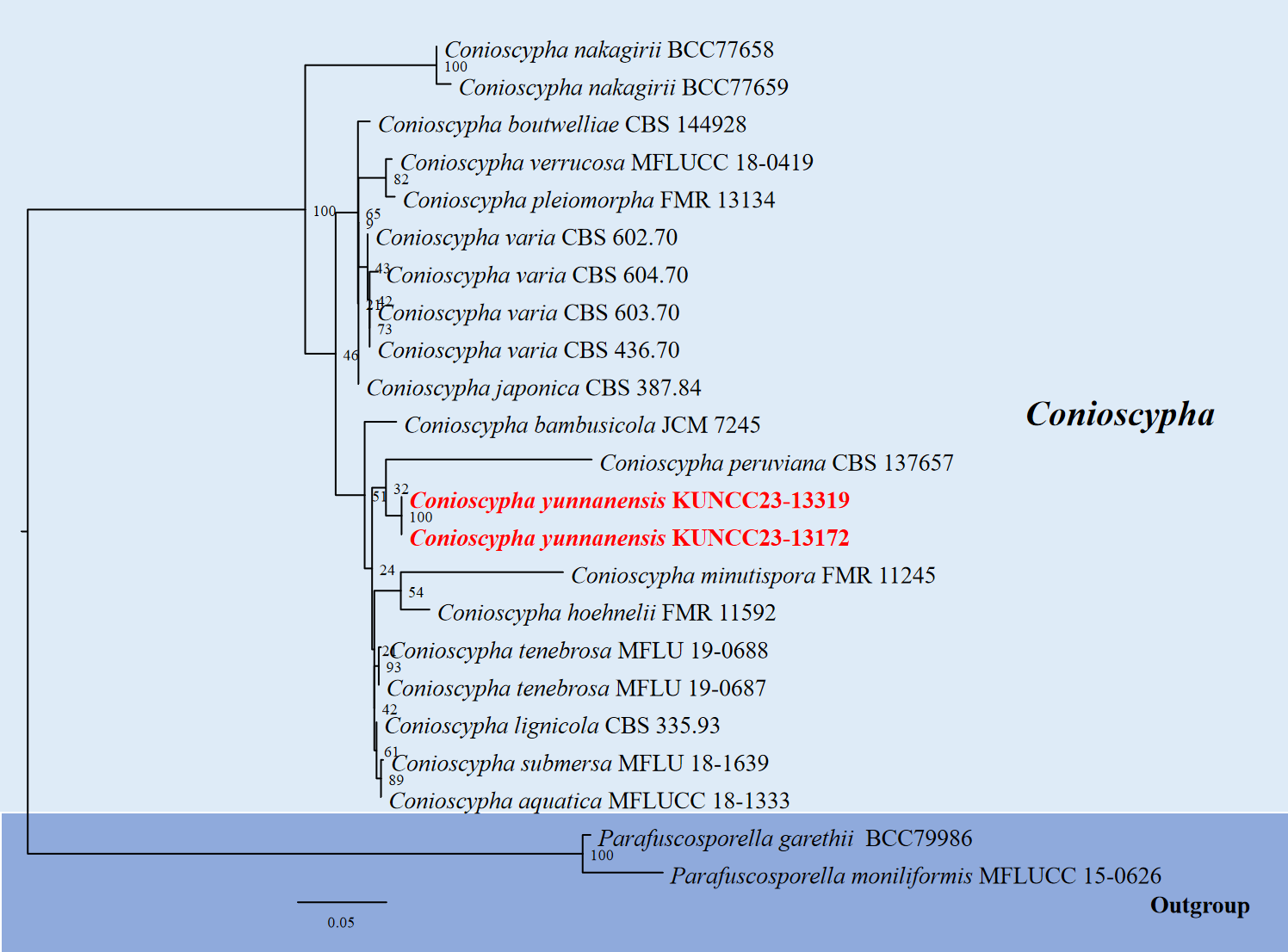


Figure 2. shows the phylogram generated from ML analysis based on LSU, sequences data for *Conioscypha*. Twenty-three strains are included in the dataset which comprised 854 characters (LSU: 854bp) after alignment. The best RAxML tree with a final likelihood value of -3208.375674 is presented. RAxML analysis yielded 252 distinct alignment patterns and 9.06% of undetermined characters or gaps. Estimated base frequencies were as follows: A = 0.227229, C = 0.256396, G = 0.325533, T = 0.190841, with substitution rates AC =0.649617, AG = 1.569871, AT = 0.855952, CG = 0.764090, CT = 7.550628, GT = 1.000000; gamma distribution shape parameter alpha = 0.568342. Phylogenetic analyses also showed that the new taxon was clustered with strong statistical support (100%ML /1.00BIPP) and formed an independent clade. Two *C. nakagirii* strains formed a basal clade in the tree and the new taxon showed the close phylogenetic relationship to *C. peruviana.* Select *Parafuscosporella garethii* (BCC79986) and *P. moniliformis* (MFLUCC 15-0626) as outgroup.


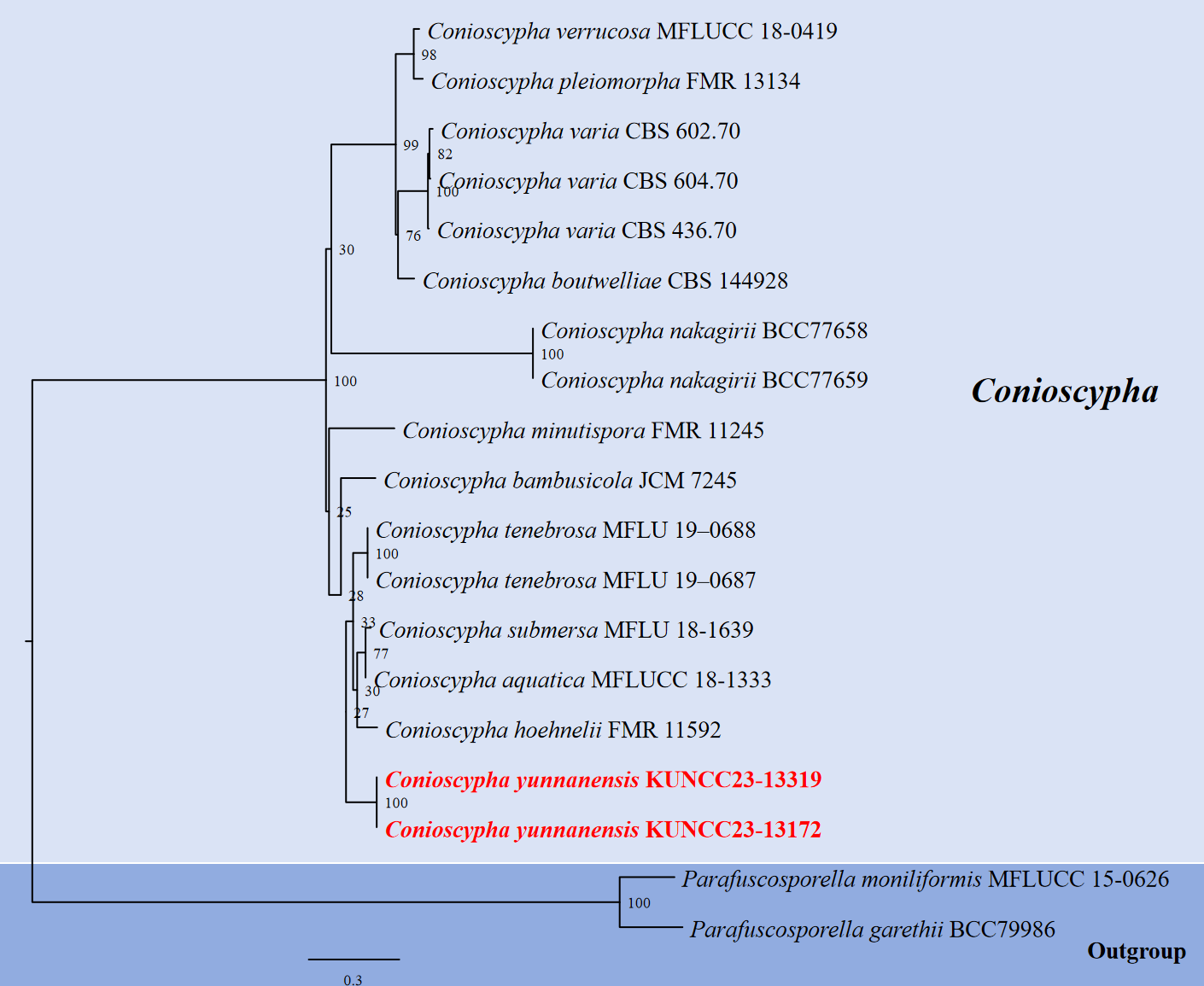


Figure 3. shows the phylogram generated from ML analysis based on ITS, sequences data for *Conioscypha*. Nineteen strains are included in the dataset which comprised 498 characters (ITS: 498bp) after alignment. The best RAxML tree with a final likelihood value of -3319.139865 is presented. RAxML analysis yielded 272 distinct alignment patterns and 4.97% of undetermined characters or gaps. Estimated base frequencies were as follows: A = 0.245218, C = 0.308163, G = 0.269128, T = 0.177491, with substitution rates AC =0.912322, AG = 1.645372, AT = 1.737754, CG = 0.698523, CT = 3.768194, GT = 1.000000; gamma distribution shape parameter alpha = 0.381285. Phylogenetic analyses also showed that the new taxon was clustered with strong statistical support (100%ML /1.00BIPP) and formed an independent clade. Select *Parafuscosporella garethii* (BCC79986) and *P. moniliformis* (MFLUCC 15-0626) as outgroup.


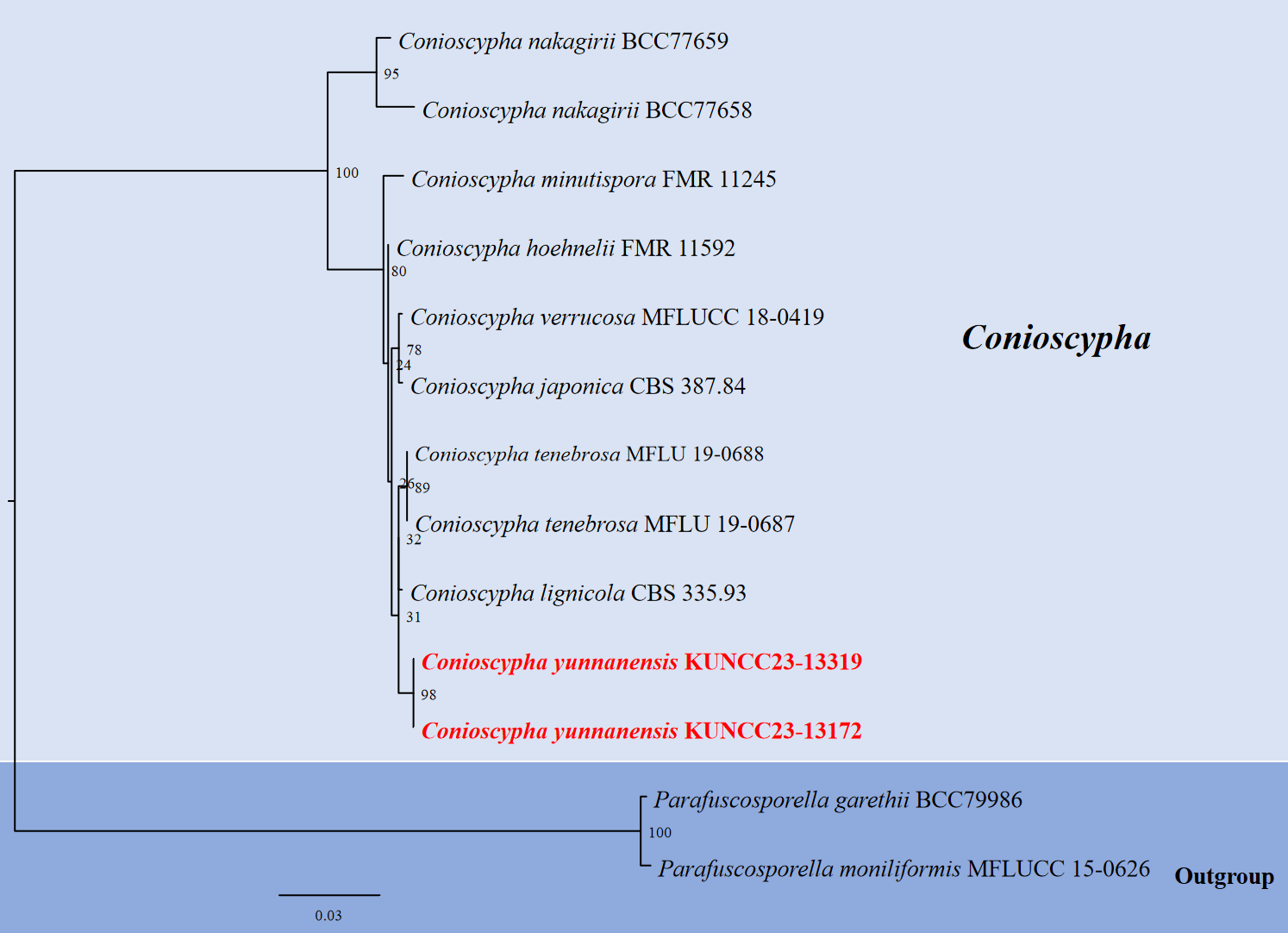


Figure 4. shows the phylogram generated from ML analysis based on SSU, sequences data for *Conioscypha*. Thirteen strains are included in the dataset which comprised 975 characters (SSU: 975bp) after alignment. The best RAxML tree with a final likelihood value of -2182.521253 is presented. RAxML analysis yielded 112 distinct alignment patterns and 4.62% of undetermined characters or gaps. Estimated base frequencies were as follows: A = 0.254446, C = 0.232277, G = 0.269667, T = 0.243610, with substitution rates AC = 2019.994576, AG = 5217.318918, AT = 1874.765192, CG = 1617.012159, CT = 9949.322810, GT = 1.000000; gamma distribution shape parameter alpha = 0.868165. Phylogenetic analyses also showed that the new taxon was clustered with strong statistical support (98%ML /1.00BIPP) and formed an independent clade. Select *Parafuscosporella garethii* (BCC79986) and *P. moniliformis* (MFLUCC 15-0626) as outgroup.


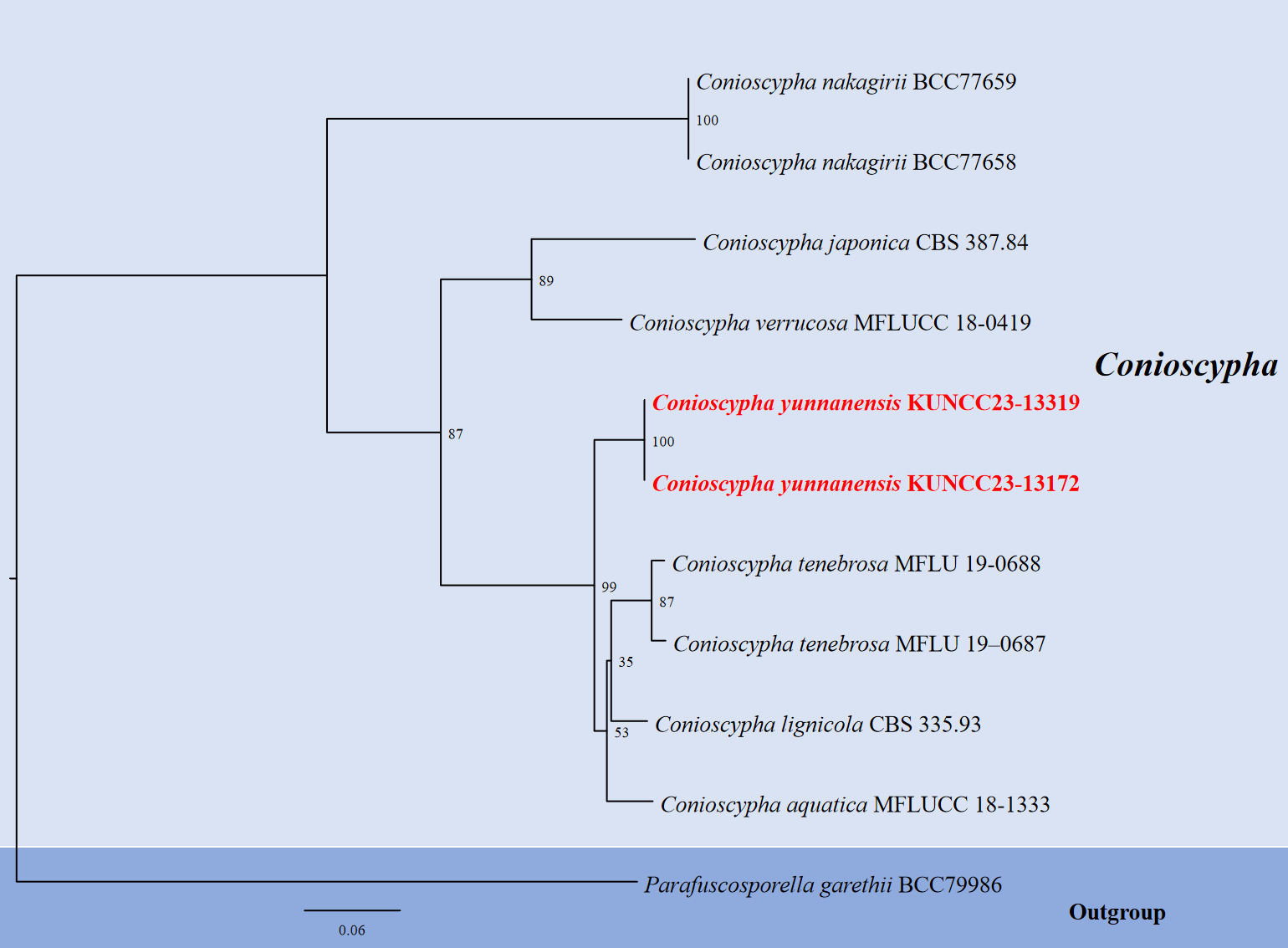


Figure 5. shows the phylogram generated from ML analysis based on *RPB2*, sequences data for *Conioscypha*. Eleven strains are included in the dataset which comprised 1007 characters (RPB2: 1007bp) after alignment. The best RAxML tree with a final likelihood value of -3787.953812 is presented. RAxML analysis yielded 263 distinct alignment patterns and 12.69% of undetermined characters or gaps. Estimated base frequencies were as follows: A = 0.224175, C = 0.286423, G = 0.298625, T = 0.190777, with substitution rates AC = 1.302209, AG = 2.210903, AT = 0.486449, CG = 1.539891, CT = 6.951168, GT = 1.000000; gamma distribution shape parameter alpha = 0.868165. Phylogenetic analyses also showed that the new taxon was clustered with strong statistical support (100%ML /1.00BIPP) and formed an independent clade. Select *Parafuscosporella garethii* (BCC79986) and *P. moniliformis* (MFLUCC 15-0626) as outgroup.


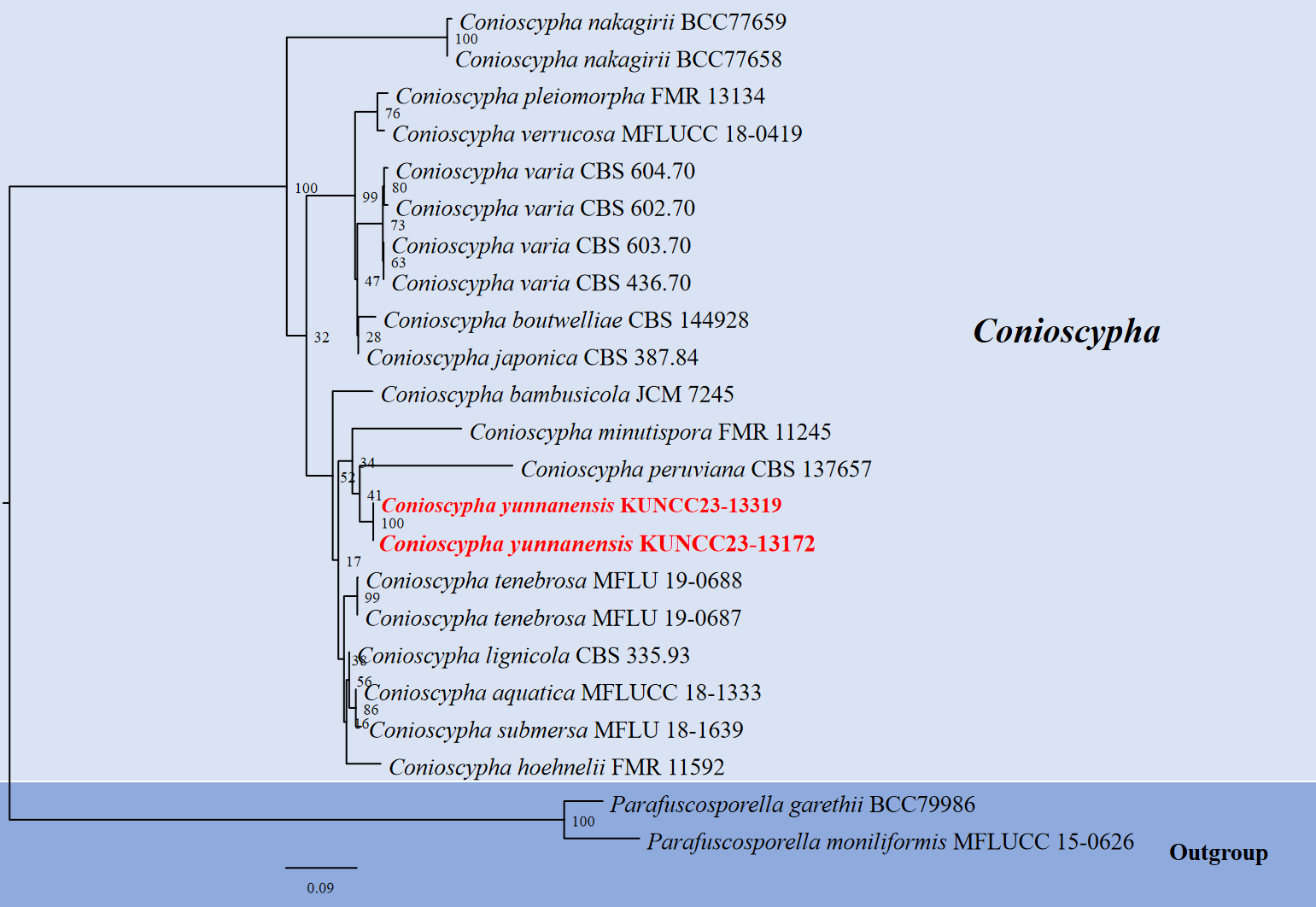


Figure 6. shows the phylogram generated from ML analysis based on combined LSU and ITS, sequences data for *Conioscypha*. Twenty three strains are included in the combined dataset which comprised 1352 characters (LSU: 854bp, ITS:498) after alignment. The best RAxML tree with a final likelihood value of -6712.378362 is presented. RAxML analysis yielded 524 distinct alignment patterns and 13.64% of undetermined characters or gaps. Estimated base frequencies were as follows: A = 0.233253, C = 0.273729, G = 0.306647, T = 0.186371, with substitution rates AC = 1.297570, AG = 2.191134, AT = 1.717332, CG = 0.961202, CT = 6.161197, GT = 1.000000; gamma distribution shape parameter alpha = 0.476308. Phylogenetic analyses also showed that the new taxon was clustered with strong statistical support (100%ML /1.00BIPP) and formed an independent clade *C.peruviana* and *C.minutispora*. Two *C. nakagirii* strains formed a basal clade in the tree and the new taxon showed the close phylogenetic relationship to *C. peruviana* and *C. minutispora.* Select *Parafuscosporella garethii* (BCC79986) and *P. moniliformis* (MFLUCC 15-0626) as outgroup.


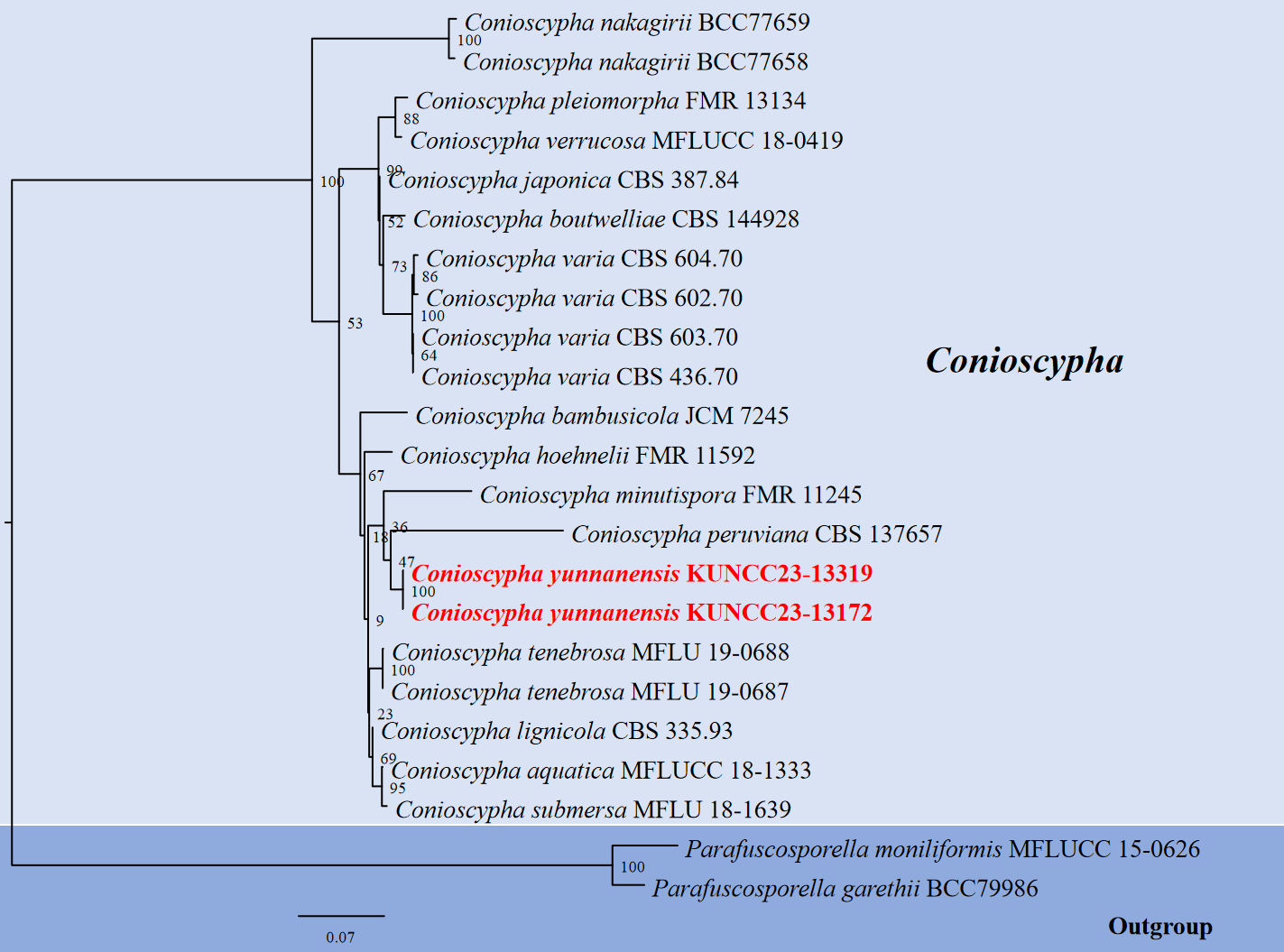


Figure 7. shows the phylogram generated from ML analysis based on combined LSU, ITS and SSU, sequences data for *Conioscypha*. Twenty three strains are included in the combined dataset which comprised 2327 characters (LSU: 854bp, ITS:498, SSU: 975bp) after alignment. The best RAxML tree with a final likelihood value of -9115.552167 is presented. RAxML analysis yielded 636 distinct alignment patterns and 27.24% of undetermined characters or gaps. Estimated base frequencies were as follows: A = 0.239832, C = 0.260861, G = 0.295166, T = 0.204141, with substitution rates AC =1.681103, AG = 3.126043, AT = 1.867844, CG = 1.376480, CT = 7.220328, GT = 1.000000; gamma distribution shape parameter alpha = 0.522693. Phylogenetic analyses also showed that the new taxon was clustered with strong statistical support (100%ML /1.00BIPP) and formed an independent clade *C.peruviana* and *C.minutispora*. Two *C. nakagirii* strains formed a basal clade in the tree and the new taxon showed the close phylogenetic relationship to *C. peruviana* and *C. minutispora.* Select *Parafuscosporella garethii* (BCC79986) and *P. moniliformis* (MFLUCC 15-0626) as outgroup.


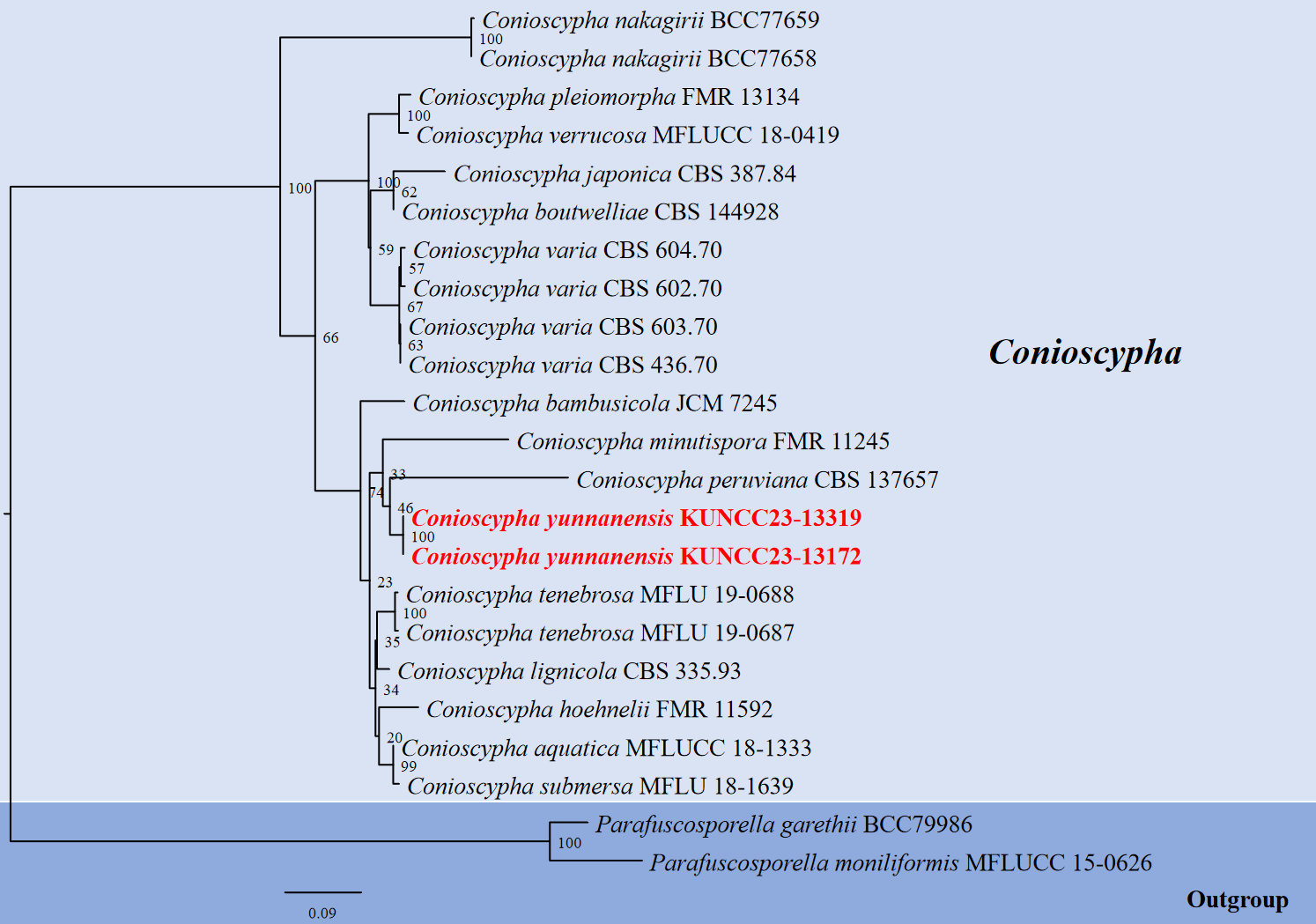


Figure 8. shows the phylogram generated from ML analysis based on combined LSU, ITS and *RPB2*, sequences data for *Conioscypha*. Twenty three strains are included in the combined dataset which comprised 2359 characters (LSU: 854bp, ITS:498, *RPB2*: 1007bp) after alignment. The best RAxML tree with a final likelihood value of -10555.781766 is presented. RAxML analysis yielded 787 distinct alignment patterns and 32.68% of undetermined characters or gaps. Estimated base frequencies were as follows: A = 0.230852, C = 0.277087, G = 0.304516, T = 0.187546, with substitution rates AC = 1.266162, AG = 2.209066, AT = 1.363118, CG = 1.140451, CT = 6.542299, GT = 1.000000; gamma distribution shape parameter alpha = 0.451063. Phylogenetic analyses also showed that the new taxon was clustered with strong statistical support (100%ML /1.00BIPP) and formed an independent clade *C.peruviana* and *C.minutispora*. Two *C. nakagirii* strains formed a basal clade in the tree and the new taxon showed the close phylogenetic relationship to *C. peruviana* and *C. minutispora.* Select *Parafuscosporella garethii* (BCC79986) and *P. moniliformis* (MFLUCC 15-0626) as outgroup.
